# A standardized herbal extract PM014 ameliorates pulmonary fibrosis by suppressing the TGF-β1 pathway

**DOI:** 10.1038/s41598-018-35320-8

**Published:** 2018-11-15

**Authors:** Kyung Hwa Kim, Sujin Lee, Hyunji Lee, Dasom Shin, Daeun Min, Miran Kim, Byeol Ryu, Hyeon Woo Kim, Hyunsu Bae

**Affiliations:** 1 Department of Physiology, College of Korean Medicine, Kyung Hee University, Seoul, 02447 South Korea; 2 Central Research Institute, Hanlim Pharm. Co. Ltd., Yongin, 17040 South Korea; 3 College of Pharmacy and Research Institute of Pharmaceutical Science, Seoul National University, Seoul, 08826 South Korea

## Abstract

Idiopathic pulmonary fibrosis (IPF) is a devastating and common chronic lung disease pathologically characterized by loss of epithelial cells and activation of fibroblasts and myofibroblasts. The etiology of IPF remains unclear and the disease pathology is poorly understood with no known efficacious therapy. PM014 is an herbal extract that has been shown to have beneficial effects in pulmonary diseases, which are likely to exert anti-inflammatory bioactions. In the present study, we observed that bleomycin (BLM) caused increased inflammatory infiltration as well as collagen deposition in lungs of mice on day 14 after treatment. Administration of PM014 suppressed BLM-induced inflammatory responses and fibrotic changes in dose-dependent manner in mice. Additionally, we provided *in vitro* evidence suggesting that PM014 inhibited TGF-β1-induced epithelial-mesenchymal transition (EMT) and fibroblast activation in alveolar epithelial cells and human lung fibroblasts from healthy donor and IPF patients. PM014 appeared to target TGF-β1 signaling via Smad-dependent pathways and p38 mitogen-activated protein kinases (MAPKs) pathways. Taken together, our data suggest that PM014 administration exerts a protective effect against lung fibrosis and highlight PM014 as a viable treatment option that may bring benefits to patient with IPF.

## Introduction

Idiopathic pulmonary fibrosis (IPF) is a chronic and devastating lung disease of unknown etiology^1^. IPF is usually characterized by exertional dyspnea and decline in lung function, only with a median survival time of 3 years from the time of diagnosis^2,3^. Currently, two drugs approved by the FDA, such as pirfenidone and nintedanib, have been considered promising therapeutic agents for slowing down the progression of the disease, mainly by changing forced vital capacity (FVC)^4,5^. Unfortunately, these drugs have some serious potential side effects and neither of these drugs have been found to effectively reverse lung fibrosis^5,6^. Thus, there is an urgent need to develop novel drugs for the treatment of IPF.

The hallmark of IPF is a chronic alveolar epithelial injury, which results in fibroblast activation, myoblast proliferation and extracellular matrix (ECM) deposition, thereby destroying lung architecture^7,8^. A plethora of mediators, including cytokines and chemokines, have been implicated in the process of lung fibrosis. Among these molecules, transforming growth factor (TGF)-β1 is one of the most potent inducers of fibrosis pathogenesis. This growth factor markedly induces epithelial-mesenchymal transition (EMT) in alveolar epithelial cells (AECs) and triggers the differentiation of fibroblasts into myofibroblasts^7^. Further, TGF-β1 can directly activate several signaling cascades, including canonical Smad-dependent and non-canonical Smad-independent signaling, which subsequently aggravate pathological changes in IPF^7,9^. Therefore, inhibition of TGF-β1 signaling may be a promising therapeutic approach in IPF.

PM014 is a novel herb extract derived from the herbal compound Chung-Sang-Bo-Ha-Tang (CSBHT), which has been widely used for treating lung disorders in traditional medicine^10^. Recently, we found that PM014 suppressed the inflammatory response in radiotherapy-induced pulmonary inflammation by regulating inflammasome activation^11^. PM014 was also documented to inhibit LPS-induced lung neutrophilia in chronic obstructive pulmonary disease (COPD)^12^. Furthermore, PM014 has been demonstrated to alleviate allergic airway inflammation and smooth muscle hypertrophy in asthma^13^.

Considering the anti-inflammatory potential of PM014 in lung diseases, we hypothesize that PM014 protects against IPF. To test this hypothesis, we induced lung fibrosis with bleomycin treatment on mice *in vivo* and also induced EMT with TGF-β1 treatment on A549 cells as well as human lung fibroblasts (HLFs) *in vitro*. We found that administration of PM014 provided the protection for mice against lung fibrosis as evidenced by reduced pulmonary fibrosis and improved lung damage. Further mechanistic studies revealed that PM014 not only repressed TGF-β1-induced EMT and fibroblast activation, but also inhibited TGF-β1 signaling pathway. Our data indicate that a novel herbal extract PM014 could be therapeutic target for treatment of pulmonary fibrosis.

## Results

### PM014 alleviates BLM-induced pulmonary fibrosis in mice

**Figure 1:**
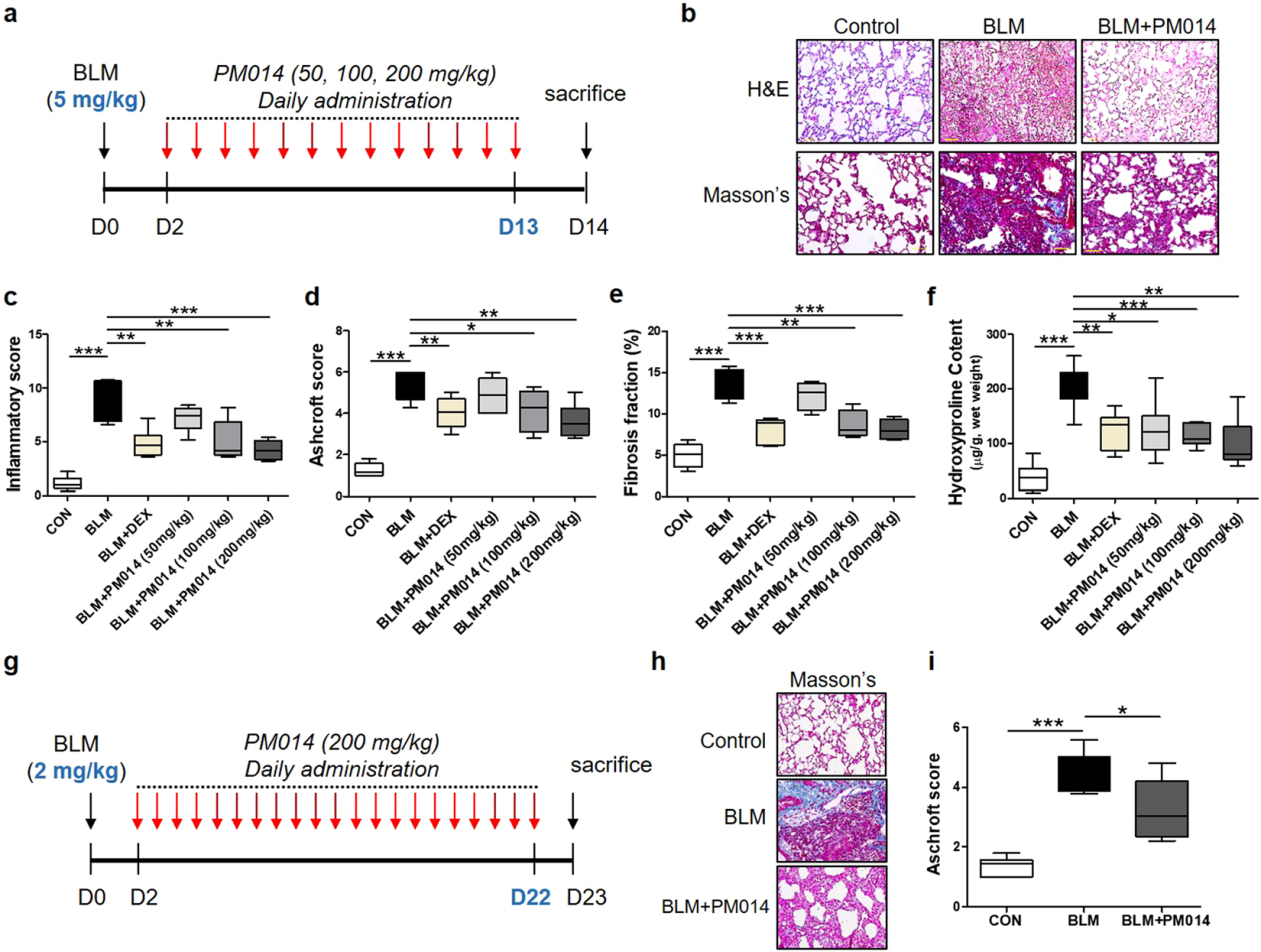
PM014 ameliorates pulmonary fibrosis induced by BLM in mice. (**a**–**f**) Mice were administered intratracheally once with BLM (5 mg/kg) at day 0 and subsequently received PM014 (50, 100, or 200 mg/kg) daily from day 2 to day 13 after BLM exposure. (**a**) The experimental schedule was represented. (**b**) Representative images of pulmonary tissues stained with hematoxylin and eosin (H&E) or Masson’s trichrome staining were shown from control, BLM, and BLM + PM014 (200 mg/kg) group. (**c**,**d**) Ashcroft scoring and morphometric analysis of collagen were performed to determine the extent of lung fibrosis. (**f**) Hydroxyproline content used to evaluate collagen content was quantified in the lung tissues. (**g**–**i**) Mice were administered intratracheally once with BLM (2 mg/kg) at day 0 and subsequently received PM014 (200 mg/kg) daily from day 2 to day 21 after BLM treatment. (**g**) The experimental schedule was represented. (**h**) Representative images of lung tissues stained with Masson’s trichrome staining are shown from control, BLM, and BLM + PM014 (200 mg/kg) group. (**i**) Ashcroft scoring system was adopted to determine the extent of lung fibrosis. Data are presented as mean ± SE, n = 4–8 for each experimental group, *P < 0.05; **P < 0.01; ***P < 0.001.

In the present study, we induced lung fibrosis by a single intratracheal administration of bleomycin as described previously^14^. In order to investigate the anti-fibrotic potential of PM014, mice challenged with BLM (5 mg/kg) were treated with three different doses of PM014 (50, 100, and 200 mg/kg) for 12 days starting at day 2 post-bleomycin (Fig. 1a). Fourteen days after BLM challenge, mice showed both inflammation and fibrosis in the lungs. When compared to control group, bleomycin produced enhanced pulmonary inflammatory responses as well as destructed lung architecture with thickened alveolar septa and collapsed alveolar spaces, as evidenced by the histological evaluation (Fig. 1b) and semi-quantitative histological analysis (Fig. 1c) of H&E stained lung sections. Additionally, mice treated with bleomycin displayed notable deposition of collagen, as indicated by not only semi-quantitative analysis using fibrotic Aschroft scoring (Fig. 1d) and quantitative histological collagen morphometry assessed by the fibrosis fraction (Fig. 1e) from Masson’s trichrome stained tissues, but also biochemical collagen quantification determined by measuring hydroxyproline (Fig. 1f). Importantly, all these BLM-induced inflammatory and fibrotic features were obviously alleviated after PM014 administration beginning 2 days after BLM treatment (Fig. 1a–f). Similar findings were obtained when comparing treatment of PM014 and dexamethasone, which can be current first-line choice for pulmonary fibrosis^15^, in mice on day 14 post-treatment of BLM (Fig. 1c,e–f).

Bleomycin is well known to induce lung damage that results in acute inflammatory response, followed by fibrotic changes which can replicate many pathological features of human pulmonary fibrosis^16,17^. Hence, many researchers have recommended to evaluate anti-fibrotic properties of any novel target drug under investigation for treatment of lung fibrosis specially when inflammation subsides (after 10–14 days after bleomycin exposure)^17,18^. Here, we conducted a deeper investigation into testing anti-fibrotic action of PM014 with preventive treatment and therapeutic treatment in order to determine whether PM014 attenuates lung fibrosis solely by inhibiting inflammatory response and/or ameliorating the progression of established lung fibrosis. Firstly, to assess the preventive effect of PM014, mice were challenged with relatively low doses of bleomycin (2 mg/kg) and treated with PM014 (200 mg/kg) for 21 days, beginning on day 2 after bleomycin treatment (Fig. 1g). Interestingly, lung fibrosis was markedly alleviated in mice treated with PM014 over the entire experimental period compared with BLM-only mice, as evidenced by Ashcroft’s fibrosis scoring (Fig. 1h,i).

Next, the therapeutic effect of PM014 was tested by two treatment schedules; mice were injected with bleomycin (5 mg/kg) and then PM014 was injected from day 7 to day 14 after BLM instillation (Fig. S1a) or from day 14 to day 21 after BLM challenge (Fig. S1b), respectively. Administration of PM014 neither beginning day 7 nor day 14 treatment induced no significant differences in fibrotic Ashcroft’s scoring (Fig. S1b,e) as well as weight loss (Fig. S1c,f) between BLM only-group and BLM + PM014 group, appearing to be lack of therapeutic effect of PM014 when it was given starting at day 7 or day 14 after BLM challenge. These data indicate that PM014 may protect against lung fibrosis, especially in the early inflammatory phase.

### PM014 improves mice survival rate and ameliorates the body weight loss in BLM-treated mice

**Figure 2:**
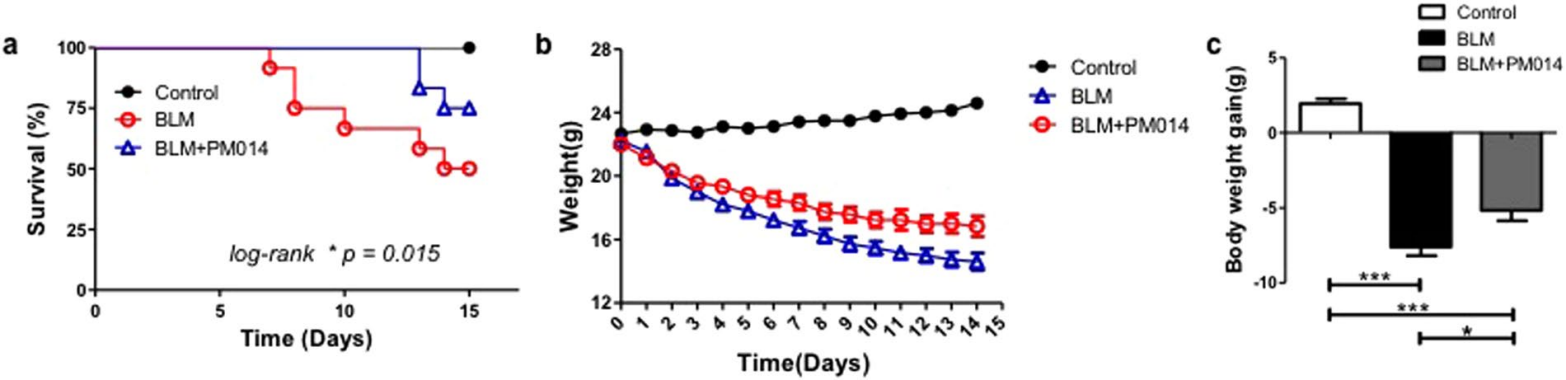
PM014 improves survival rate and ameliorates weight loss after BLM instillation. Mice were treated with BLM (5 mg/kg) intratracheally on day 0. PM014 (200 mg/kg) was administrated orally once a day, beginning on day 2 until day 13 after BLM treatment. (**a**) Survival curve was shown. Data was represented as percentage of survival at each time point. Log-rank test, *P = 0.015, n = 12 for each experimental group. (**b**,**c**) (**b**) Body weight was measured every day. (**c**) Body weight gain over 15 days was calculated. n = 15 for each experimental group, *P < 0.05; ***P < 0.001, Data are presented as mean ± SE.

Next, to assess the protective effects of PM014, mice were treated with bleomycin at a dose of 5 mg/kg to induce lung damage and mortality. The changes of body weight were measured to assess the pulmonary toxicity induced by bleomicin. As shown in Fig. 2a, the mice treated with bleomycin began to die at day 7 and cumulative mortality was 50% at day 14 in BLM mice. Importantly, the survival rate was significantly improved by BLM + PM014 (200 mg/kg) mice compared with BLM-only mice. In addition, post-treatment with PM014 ameliorated the body weight loss of BLM-treated mice (Fig. 2b,c).

### PM014 possesses an anti-inflammatory function in lung fibrosis induced by BLM

**Figure 3:**
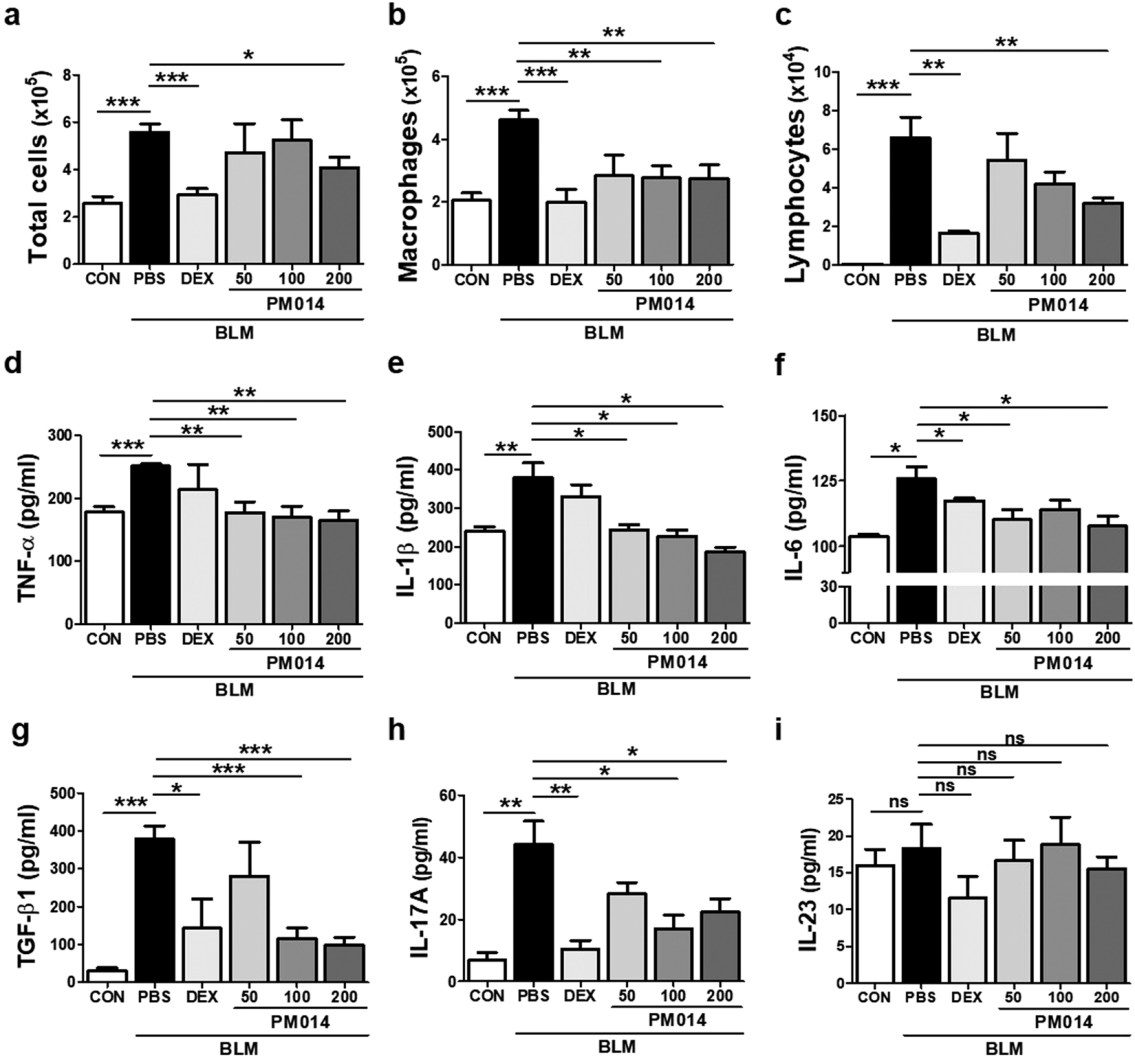
PM014 inhibits pulmonary inflammation in lung fibrosis induced by BLM. Mice were injected with BLM (5 mg/kg) on day 0, followed by PM014 (50, 100, or 200 mg/kg) daily for consecutive 12 days, beginning on day 2 after BLM administration. (**a**–**c**) Bronchoalveolar lavage fluid (BALF) analysis was carried out by counting the number of total inflammatory cells, macrophages, and lymphocytes. (**d**–**i**) The level of pro-inflammatory cytokines from lung tissues of mice were analyzed by ELISA. The investigated cytokines were TNF-α, IL-1β, IL-6, TGF-β1, IL-17A, and IL-23. NS indicates no significant differences were found among groups. Data are presented as mean ± SE, n = 4–11, *P < 0.05; **P < 0.01; ***P < 0.001.

Since BLM can induce influx of inflammatory cells into lung during early inflammatory phase^17,18^, we analyzed the influx of important inflammatory cells into the lungs by performing bronchoalveolar lavage fluid (BALF) counting analysis in BLM mice on day 14 after BLM instillation (5 mg/kg). As shown in Fig. 3a–c, increased infiltration of total cells, macrophages, and lymphocytes in lungs of BLM mice was observed, which is consistent with previous observations on elevated lung inflammation from BLM mice through day 14 after BLM treatment^19^. PM014 significantly reduced the number of infiltrated cells in the BALF of BLM-treated mice in a concentration-dependent manner. Similarly, dexamethasone which has been known to have powerful anti-inflammatory activity blocked the accumulation of inflammatory cells in the lungs of BLM-treated mice.

Crosstalk between cytokines and fibroblasts during lung fibrosis is a growing research interest^20^. Hence, in this study, we sought to examine the expression of a variety of inflammatory cytokines, such as tumor necrosis factor (TNF)-α, interleukin (IL)−1β, IL-6, transforming growth factor (TGF)-β, IL-17A, and IL-23 (Fig. 3d–i). Marked increases in production of TNF-α, IL-1β, IL-6, TGF-β, and IL-17A were seen on day 14 after BLM exposure, whereas there were no significant differences in the levels of IL-23 on BLM-treated mice when compared with control mice. Nonetheless, we observed that PM014 exhibited a reduction of the expression level of TNF-α, IL-1β, IL-6, TGF-β, and IL-17A in the lungs of BLM-treated mice. Markedly, PM014 administration (200 mg/kg) induced greater or similar anti-inflammatory effect on pro-inflammatory cytokines than dexamethasone in BLM-challenged mice. However, no significant differences were detected when the expression level of IL-23 between the groups was compared. Our observations indicate that PM014 significantly suppressed BLM-mediated production of inflammatory cytokines.

### PM014 represses EMT in lung fibrosis induced by BLM

**Figure 4:**
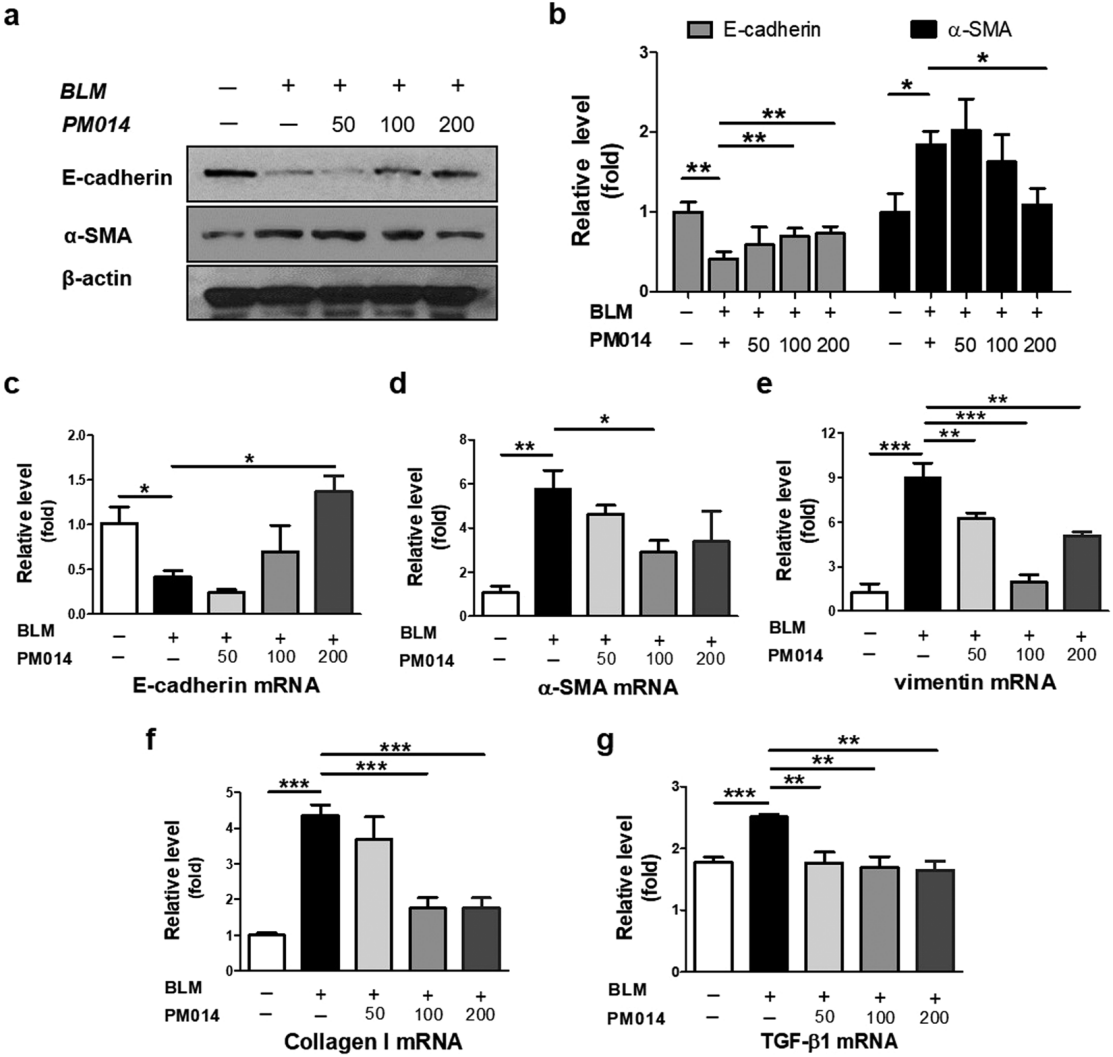
PM014 represses EMT and fibroblast activation in pulmonary fibrosis in mice. Mice were intratracheally injected with a single dose of BLM (5 mg/kg) and subsequently treated with different doses of PM014 (50, 100, or 200 mg/kg) for 12 days, starting at day 2 after BLM exposure. (**a**) Western blotting analysis of E-cadherin and α-SMA in the lung tissues of mice. (**b**) Densitometric analysis, represented as the percentage of E-cadherin and α-SMA compared to levels in control group. (**c**–**g**) Quantitative real-time qPCR analysis of E-cadherin, α-SMA, vimentin, type I collagen, and TGF-β1 to measure the mRNA levels in the lung tissues derived from each group. The cropped gels are used in the figure, and uncropped gels are presented in Fig. S2. Data are presented as mean ± SE, n = 3–4, *P < 0.05; **P < 0.01; ***P < 0.001.

Epithelial-mesenchymal transition (EMT), a process whereby differentiated epithelial cells acquire mesenchymal phenotypes during fibrotic processes, is increasingly recognized as an important phenomenon in lung fibrosis progression^7,8^. Thus, we first investigated whether the anti-fibrotic effect of PM014 was mediated via inhibition of EMT. As shown in Fig. 4a,b, bleomycin (5 mg/kg) seemed to induce myofibroblast activation, which was associated with decreased expression of E-cadherin (epithelial marker) and increased expression of α-smooth muscle actin (SMA) (mesenchymal marker). On the contrary, PM014 intervention for 12 days, beginning at day 2 after BLM treatment, resulted in inhibition of EMT in BLM-treated mice, as determined by the changes in expression of EMT markers. Likewise, our real-time qPCR analysis further demonstrated that BLM down-regulated E-cadherin (Fig. 4c) and up-regulated α-SMA (Fig. 4d) and vimentin (mesenchymal marker) (Fig. 4e) expression, when compared with control mice. Nevertheless, PM014 treatment enhanced the expression of E-cadherin and reduced the expression of α-SMA and vimentin, when compared with BLM alone treated mice (Fig. 4c–e), indicating the inhibitory effect of PM014 in bleomycin-induced EMT. Importantly, endogenous mRNA level of type I collagen which is the major fibrous collagen synthesized during lung fibrosis was obviously increased after bleomycin treatment (Fig. 4f). Administration of PM014 markedly inhibited increased expression of collagen induced by BLM exposure. In addition, we assessed endogenous mRNA level of TGF-β1 in BLM-treated mice because TGF-β1 is one of the main signaling molecules that stimulates EMT during pulmonary fibrosis^7,9^. As shown in Fig. 4g, up-regulated TGF-β1 in BLM-challenged mice was reduced by administration of PM014 in concentration-dependent manner.

### PM014 blocks TGF-β1-induced EMT in alveolar epithelial A549 cells

**Figure 5:**
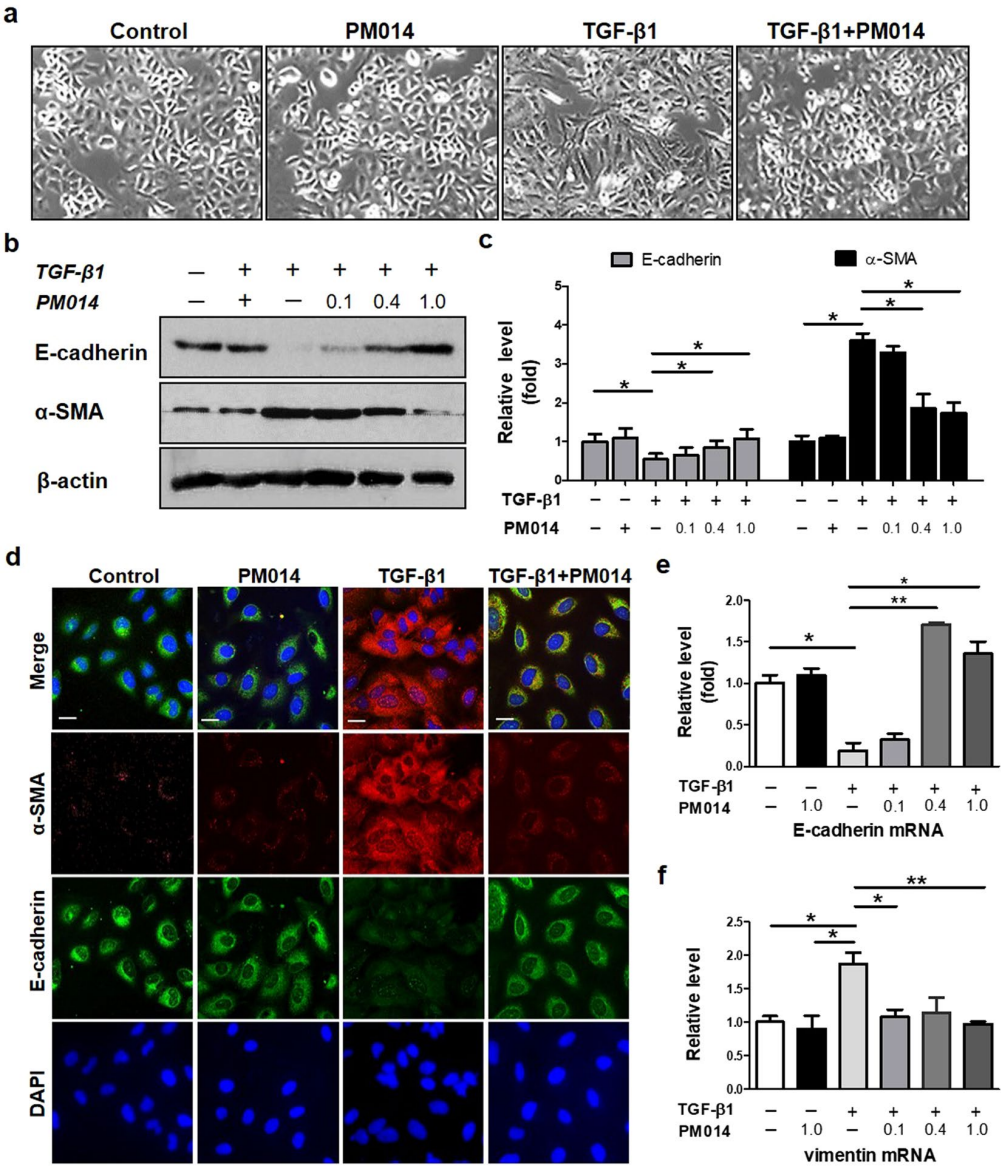
PM014 suppresses TGF-β1-induced EMT and fibroblast activation in human alveolar epithelial A549 cells. A549 cells were treated with TGF-β1 (20 ng/ml) and PM014 (0.1, 0.4, 1.0 mg/ml) for 48 h; 0.05% dimethylsulfoxide (DMSO) was used as a vehicle control for stimulation. (**a**) The morphological changes were imaged using phase contrast microscopy (shown at 200x magnification). (**b**–**d**) The protein levels of E-cadherin and α-SMA were evaluated by western blotting (**b**) and subsequently analyzed by densitometry (**c**) and immunofluorescence (**d**). Data are presented relative to an average of the controls. (**e**,**f**) Real-time qPCR analysis of E-cadherin and vimentin in A549 cells treated with TGF-β1 and PM014. The cropped gels are used in the figure, and uncropped gels are presented in Fig. S3. Data are presented as the mean ± SE, n = 3–4, *P < 0.05; **P < 0.01, scale bar, 10 μm.

We further evaluated whether the anti-fibrotic effect of PM014 was mediated via inhibition of EMT using *in vitro* model of EMT. TGF-β1 is well known to initiate and maintain EMT in various biological system including alveolar epithelial cells (AECs)^21^. Here, we induced EMT in A549 cell, the most popular cell lines of human alveolar epithelial type II cells, with treatment of TGF-β1 for 48 h. Indeed, TGF-β1-induced morphological alteration in A549 was clearly detected, from oval epithelial cells to spindle shaped fibroblast-like cells (Fig. 5a). Correlating in *in vivo* results with BLM-induced mice, decreased expression of E-cadherin and increased expression of α-SMA were detected (Fig. 5b,c). PM014 intervention markedly blocked the spindle-like mesenchymal morphology phenotype of EMT in A549 cells stimulated by TGF-β1 (Fig. 5a). As observed, PM014 treatment increased the expression of the epithelial marker E-cadherin and repressed the expression of the mesenchymal marker α-SMA in TGF-β1-treated A549 cells in a dose-dependent manner (Fig. 5b,c). Likewise, immunofluorescence study further demonstrated that PM014 treatment enhanced the expression of E-cadherin and reduced the expression of α-SMA in A549 cells compared with those of TGF-β1 treatment alone (Fig. 5d). Apparently, PM014 dose-dependently increased mRNA level of endogenous E-cadherin and reduced the level of vimentin in TGF-β1-treated epithelial cells (Fig. 5e,f).

### PM014 targets canonical Smad and non-canonical p38 signaling activated by TGF-β1 in alveolar epithelial cells and lung fibroblasts

**Figure 6:**
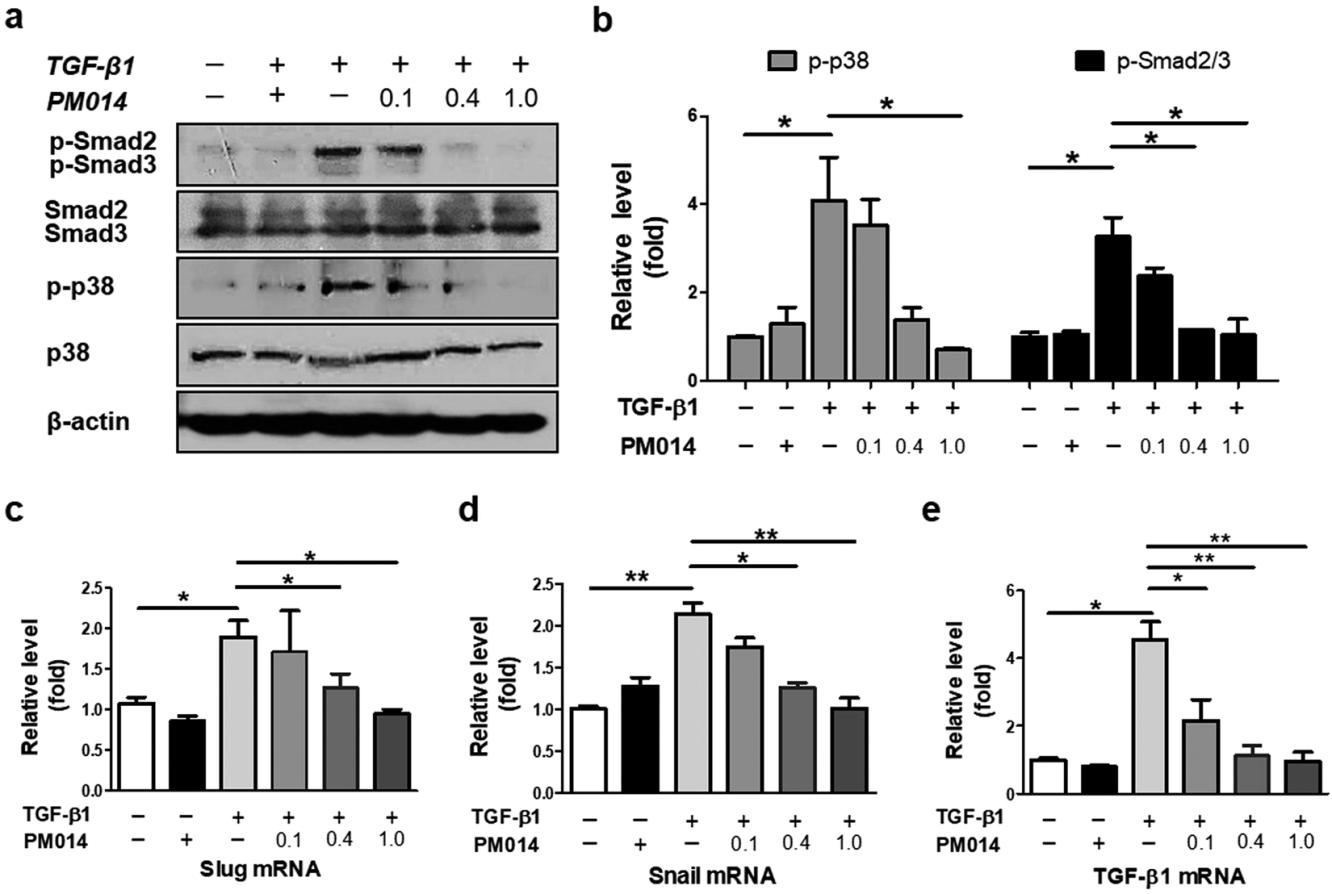
PM014 suppresses TGF-β1 pathway through inactivating canonical and non-canonical signaling in A549 cells. Human epithelial alveolar A549 cells were stimulated with TGF-β1 (20 ng/ml) and PM014 (0.1, 0.4, 1.0 mg/ml) for 48 h. (**a**) Western blotting analysis of phosphorylation levels of Smad2 and Smad3 and p38 MAPK. (**b**) Densitometry of the immune blot was analyzed quantitatively. Expression of p-Smad2/3 and p-p38 is represented as a percentage compared to that of the control DMSO-treated group. (**c**–**e**) Real-time qPCR was performed to examine the mRNA levels of Slug, Snail, and TGF-β1. The cropped gels are used in the figure, and uncropped gels are presented in Fig. S4. Data are presented as mean ± SE, n = 3–4, *P < 0.05; **P < 0.01.

In order gain insight into the mechanisms underlying PM014 inhibition of EMT and fibroblast activation during fibrotic processes, we first examined whether PM014 could modify Smad-dependent pathway, which is well known as canonical signaling activated by TGF-β1^22^. Indeed, TGF-β1 induced increased phosphorylation of Smad2 and Smad3 in A549 cells when compared with control cells (Fig. 6a,b). Whereas, co-treatment with PM014 and TGF-β1 markedly decreased the phosphorylation of Smad2 and Smad3 compared with TGF-β1 treated A549 cells. Next, we tested the effects of PM014 on the activation of p38 mitogen-activated protein kinases (MAPKs), which has been considered as key player influencing of non-canonical Smad-independent pathway^23^. Interestingly, PM014 inhibited TGF-β1-induced phosphorylation of p38 MAPK without altering the total expression of levels in A549 cells (Fig. 6a,b).

In order to determine whether PM014 is capable of exerting anti-fibrotic effects on fibroblast cells, we assessed activation of p38 MAPK as well as Smad2 and Smad3 in lung fibroblasts from healthy donor and IPF patients (Fig. S5). Remarkably, compared with control group, TGF-β1 treatment activated not only Smad2 and Smad3, but also p38 MAPK with a concomitant expression of α-SMA in both donor and IPF fibroblasts, while PM014 co-treatment resulted in a dose-dependent suppression of TGF-β1 signaling pathway stimulating EMT.

It is well documented that TGF-β1 elicits EMT via induction of transcriptional repressors, including Snail and Slug^24^. As expected, mRNA levels of Snail and Slug were significantly increased in TGF-β1-stimulated A549 cells when comparing with control cells (Fig. 6c,d). However, PM014 co-treatment in a concentration-dependent manner inhibited expression of Snail and Slug induced by TGF-β1 in A549 cells. Surprisingly, we observed that PM014 down-regulated endogenous mRNA level of TGF-β1 transcripts induced by exogenous TGF-β1 treatment in A549 cells (Fig. 6e), which may be associated with autocrine TGF-β1 signaling^25,26^.

Consistent with previous result^27^, the addition of exogenous TGF-β1 increased mRNA level of type I collagen on human lung fibroblasts. We observed increased collagen mRNA in donor (about 2.4-fold) and IPF patients (about 4.8-fold) fibroblasts after TGF-β1 treatment, when compared with control group (Fig. S7). On the contrary, co-treatment with PM014 significantly prevented TGF-β1-induced collagen production in fibroblasts from IPF patients, not from healthy donor. Only a weak trend toward significance (*p* = 0.151) was found when comparing TGF-β1 treated and TGF-β1 + PM014 (1.0 mg) treated donor HLFs. Further studies are needed to clarify biological action of PM014 on fibroblast in lung fibrosis.

### PM014 inhibits migration in TGF-β1 stimulated A549 cells and human lung fibroblasts

**Figure 7:**
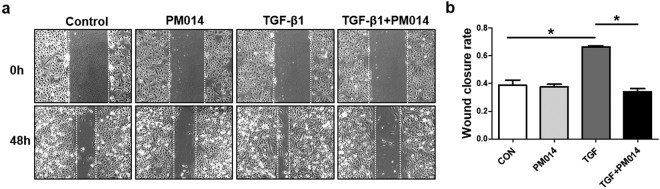
PM014 inhibits cell migration in EMT induced by TGF-β1 in alveolar epithelial A549 cells. A549 cells were treated with TGF-β1 (20 ng/ml) and PM014 (0.4 mg/ml) for 48 h. (**a**) Representative images of wound healing scratch assay in A549 cells. (**b**) The wound closure rate measuring the migration rate of A549 cells, represented as the percentage of wound closure. Data are presented as mean ± SE, n = 4, *P < 0.05.

Cell migration is one of key phenotypic changes of lung fibrosis, which appears to be key process for developing an aggressive phenotype^28^. As shown in Fig. 7, TGF-β1 treatment caused enhanced cell migration activity when comparing with control A549 cells. However, co-treatment of PM014 and TGF-β1 in A549 cells significantly inhibited cell migration compared with the cells treated with TGF-β1 only, suggesting an inhibitory effect of PM014 on cell migration. Furthermore, we also evaluated whether PM014 was capable of inhibiting fibroblast migration in lung fibroblasts from healthy donor and IPF patient (Fig. S8). Co-treatment of PM014 with TGF-β1 induced remarked reduction of the migration of healthy donor fibroblasts (Fig. S8a) and IPF fibroblasts (Fig. S8b) compared with fibroblasts treated with TGF-β1 only, respectively. Taken together, these observations have led to the suggestion that PM014 may act inhibitory agent during lung fibrosis, in particular, by suppressing cell migration.

## Discussion

Here, we demonstrated for the first time the effects of PM014 against lung fibrosis with i*n vivo* and *in vitro* studies. When compared with BLM-treated mice, PM014 effectively reversed the destruction of lung architecture, inhibited the influx of inflammatory cells into the lung and suppressed production of a variety of pro-inflammatory cytokines. Apparently, PM014 induced up-regulation of epithelial markers and down-regulation of mesenchymal markers *in vivo* and *in vitro*, suggesting that PM014 may target EMT process in the pathogenesis of pulmonary fibrosis. We also demonstrated the potential utility of PM014 as a signaling pathway inhibitor that can block TGF-β1 signaling by inactivating Smad-dependent and Smad-independent p38 MAPK signaling in alveolar epithelial cells and lung fibroblasts.

An herbal formula, PM014 is composed of 7 components of CSBHT (Chung-Sang-Bo-Ha-Tang), which has been widely used in Korea for the treatment of pulmonary diseases^10^. Our previous study showed that PM014 possessed an anti-inflammatory role in radiation-induced pulmonary inflammation, mainly due to the inhibition of inflammasome activation, with inducing a global improvement of lung function^11^. We also found that PM014 intervention effectively decreased several pro-inflammatory cytokines and chemokines which were elevated in pulmonary inflammation induced by radiation. These findings are consistent with an earlier report in which PM014 blocked the influx of inflammatory cells and decreased pro-inflammatory cytokines, such as TNF-α and IL-6, in a murine model of COPD^12^. In the present study, lung fibrosis induced by BLM (5 mg/kg) displayed increased infiltration of inflammatory cells and production of pro-inflammatory cytokines in mice at day 14 after bleomycin injury (Fig. 3). A handful of studies suggest that collagen-producing activated fibroblasts or myofibroblasts are associated with inflammatory cells including macrophages and neutrophils. Growing evidence points toward a broader connection between inflammation and lung fibrosis, despite the controversies. Indeed, lung injury is known to activate macrophages, which subsequently promote inflammation by interacting with other inflammatory cells and ultimately stimulate fibroblasts to proliferate and synthesize collagen leading to fibrosis^29,30^. The pro-inflammatory cytokines including IL-1β, TNF-α, and TGF-β1 released from recruited inflammatory cells are capable of inducing fibrosis as pro-fibrotic factors^31^. In particular, TGF-β1 plays an important role on not only inducing EMT, but also stimulating of differentiation of IL-17 producing helper T (Th17) cells, further exacerbating inflammatory responses during lung fibrosis. In the present study, we observed enhanced expression levels of these pro-inflammatory cytokines, such as TNF-α, IL-1β, IL-6, TGF-β1, and IL-17A from lungs of BLM mice on day 14 after BLM exposure, while daily administration of PM014 effectively prevented BLM-induced secretion of cytokines when it was injected on day 2 after BLM treatment (Fig. 3d–h). However, no significant difference in the expression levels of IL-23 was observed in the lungs of BLM-treated mice compared with control mice (Fig. 3i). IL-23 is well known to key regulator to promote the development of IL-17-procuding helper T cells^31^. Interestingly, recent studies reported that IL-17 is produced not only by Th17 cells, but also by other cells including CD8^+^ T cells and neutrophils^32^, further suggesting that there are still gaps in our understanding the role of Th17 immune response in pulmonary fibrosis. Taken together, our observations have led to suggestion that PM014 may inhibit initiation and progression of the inflammatory response in lung fibrosis through antagonism of pro-inflammatory cytokines produced by inflammatory cells. Future studies exploring how PM014 directly acts in an anti-inflammatory way in lung fibrosis may provide mechanistic insight into the therapeutic effects of PM014.

IPF is a complex lung disease with a poor diagnosis and few treatment options, reflecting our limited knowledge of lung fibrosis pathophysiology. Generally, three developing phases in lung fibrosis were reported on mice given by a single intratracheal injection of bleomycin^33^: during the first three days after treatment, an inflammatory phase is triggered; at around 7–14 days, inflammation has been subsided and fibrosis is detected; after 15 days, the fibrosis is developed and persists up to 4 weeks post-BLM. In the present study, we observed that enhanced inflammation in the lungs of mice treated with bleomycin at dose of 5 mg/kg at day 14 post-BLM, indicating inflammation does not completely resolve during the development of fibrotic response. Nonetheless, PM014 in dose-dependent manner suppressed these BLM-mediated inflammatory responses, including infiltration of inflammatory cells in BAL fluids (Fig. 3a–c) and production of pro-inflammatory cytokines in lung (Fig. 3d–i). The anti-inflammatory effect of PM014 was confirmed by histological analysis, as evidenced by semi-quantitative quantification in H&E stained lung tissues (Fig. 1b,c). Therefore, we speculated that the anti-inflammatory activity of PM014 in this model may be partially associated with inhibiting infiltrated inflammatory cells after lung injury. In this case, further studies about clarifying anti-inflammatory potential of PM014 during early inflammatory phase (around day 1–7 after BLM instillation) may be of great interest.

It is generally recommended to test anti-fibrotic effect of investigational products after acute inflammatory phases (at least 7–10 d after bleomycin treatment), in particular in order to confirm the therapeutic effect of drugs for treatment of lung fibrosis^17,18^. Recognizing the need for practical consideration, thus, we examined anti-fibrotic effect of PM014 on BLM mice with different therapeutic treatment schedules. One group received with 5 mg/kg of BLM was administered once daily with PM014 (200 mg/kg) from day 7 to day 14 after BLM exposure (Fig. S1a–c). The second group given with 5 mg/kg of BLM was treated once daily with PM014 (200 mg/kg) from day 10 to day 21 after BLM challenge (Fig. S1d–f). Unexpectedly, no significant anti-fibrotic effect was detected from PM014 + BLM mice in any therapeutic treatment group compared with BLM mice, as assessed by fibrotic Aschroft scoring (Fig. S1b,e) as well as weight loss associated with bleomycin (Fig. S1c–f). Thus, we speculated that PM014 may play preventive role in pulmonary fibrosis before fibrotic phase. Interestingly, it was recently reported that a 5-fold higher dose is required to achieve therapeutic effect than that of preventive effect^34^. Further studies are needed to test the therapeutic efficacy of PM014 with different doses in established lung fibrosis.

A growing evidence indicates that EMT is involved in the development of lung fibrosis although the details are still unclear^7,8^. Many histological studies showed inconsistent results in the role of EMT during lung fibrosis, mainly due to lack of available method to detect dynamic and transient state of EMT as well as marker to clearly evaluate mesenchymal phenotypes^35,36^. In this study, we found that PM014 significantly reversed EMT phenotypic changes from *in vivo* BLM-treated mice (Fig. 4) and *in vitro* TGF-β1-treated epithelial cells (Fig. 5) or fibroblasts (Fig. S5). Surprisingly, PM014 treatment inhibited the upregulation of TGF-β1 expression in epithelial EMT-induced by TGF-β1 (Fig. 6e). Moreover, PM014 appeared to block TGF-β1-mediated EMT via suppression of transcriptional factors including Snail and Slug which are known to key regulators of TGF-β1-induced EMT^37,38^. Thus, we hypothesized that PM014 exerted its anti-fibrotic effect, in particular, via inhibiting EMT or inactivating TGF-β1 signaling.

In conclusion, this study firstly demonstrates that PM014 has anti-inflammatory and anti-fibrotic actions in lung fibrosis induced by bleomycin. Additionally, mechanistic *in vitro* studies reveal that PM014 inhibits TGF-β1 signaling and ameliorates phenotypic changes of EMT and myofibroblast activation. Hence, we propose PM014 has a great potential to be alternative treatment for pulmonary fibrosis.

## Methods

### Animals

Seven-week-old male C57BL/6 mice were purchased from Charles River (Orient Bio, Seongnam, South Korea). All animals were housed in a controlled environment (12/12 h light/dark cycle; 22 ± 1 °C) and given ad libitum access to food and water. All the animal experiments were approved by the Animal Experimental Ethics Committee of the Kyung Hee University (permit number: KHUASP (SE)-16-033) and were performed according to the local guidelines.

### Cell culture

Human alveolar epithelial A549 cells and primary human lung fibroblasts were purchased from ATCC (Manassas, VA, USA) and Lonza (Walkersville, MD, USA), respectively. A549 cells were cultured in Dulbecco’s modified Eagle’s medium (DMEM, 4.5 g/L glucose) (Life Technologies, USA) supplemented with 10% fetal bovine serum. Primary human lung fibroblasts were cultured in Fibroblast Cell Basal Medium and growth supplements from Lonza.

### Reagents and antibodies

Recombinant human TGF-β1 protein was purchased from R&D Systems (Minneapolis, MN, USA). BLM was acquired from Nippon Kayaku (Tokyo, Japan). Dulbecco’s modified Eagle’s medium (DMEM), fetal bovine serum (FBS), penicillin, and streptomycin were purchased from Life Technologies (Grand Island, NY, USA). Antibodies against phospho-Smad2/3, Smad2/3, phospho-p38, and p38 were obtained from Cell Signaling Technology (Boston, MA, USA). Antibodies against E-cadherin and α-SMA and β-actin were purchased, respectively, from Abcam (Cambridge, MA, USA) and Santa Cruz Biotechnology (Dallas, TX, USA). Horseradish peroxidase-conjugated anti-rabbit and anti-goat secondary antibodies were obtained from Cell Signaling Technology and Santa Cruz Biotechnology, respectively.

### Preparation of PM014

Medical plants of the seven species that constitute PM014 were prepared by Hanlim Pharm Co. LTD (Yongin, Korea) as described previously^11^. Briefly, each herb was mixed, dissolved, and extracted using a reflex condenser presented in Supplementary Table 1. Undesirable materials were removed by filtering (Sigma, St. Louis, USA). Then, the solvent was evaporated under vacuum and dried extract powder was collected as PM014. 20 mg/ml of PM014 was dissolved in HPLC-graded methanol and filtered using Minisart RC15 (Sartorius Stedium Biotech, Germany). For administration, PM014 was further diluted in PBS at different concentration. The quantities of standard materials in 1 g of the final PM014 extract were: Paeoniflorin >0.43 mg, Schizandrin >0.12 mg, Baicalin >7.26 mg, and Amygdalin >2.48 mg. Quantification of standard materials in PM014 was performed by high performance liquid chromatography (HPLC) system.

### Bleomycin-induced pulmonary fibrosis mouse model

Pulmonary fibrosis was induced using bleomycin treatment (5 mg/kg or 2 mg/kg) as described previously^14^. Briefly, male C57BL/6 mice were randomly divided into four groups: a Control group, a bleomycin (BLM) group, a BLM + PM014 group and a BLM + DEX (dexamethasone, 0.45 mg/kg). In the BLM + PM014 group, mice in three subgroups were treated with PM014 at a dose of 50, 100, or 200 mg/kg. At day 0, the mice were lightly anesthetized using isoflurane, and BLM was dissolved in 40 µl of PBS and administered intratracheally as previously described^39^. To test preventive effect, on day 2 after BLM challenge PM014 was administered orally daily for 12 days (inflammatory phase) on mice given 5 mg/kg of BLM and 21 days (fibrotic phase) on mice received 2 mg/kg of BLM, respectively. Further, to test therapeutic effect of PM014, mice were examined under two different administration schedules. One group received with 5 mg/kg of BLM was administered once daily with PM014 (200 mg/kg) from day 7 to day 14 after BLM challenge. The second group given with 5 mg/kg of BLM was treated once daily with PM014 (200 mg/kg) from day 10 to day 21 after BLM exposure. All mice were killed 24 h after the last injection and the lungs were harvested for the assays described below.

### Chromatography conditions

A high performance liquid chromatography (HPLC) system was equipped with variable wavelength detector (VWD, SHMADZU, Japan). Chromatographic separation was performed on C18 column (250 mm × 4.6 mm, 5.0 μm; YMC Co., Ltd., Kyoto, Japan) at 30 °C. The mobile phases were acetonitrile (A) and 0.1% H_3_PO_4_ in H_2_O (B), with the following gradient (A to B): 10–90% (0–2 min), 13–87% (2.1–6.5 min), 16.5–83.5% (7.5–8.5 min), 80-20% (21 min), 100-0% (21.1-24 min), 10–90% (24.1–26 min). The flow rate and injection volume were 1.5 ml/min and 50 μl, respectively. The chromatogram was recorded at 280 and 232 nm.

### Analysis of bronchoalveolar lavage fluid

After all animal were sacrificed, bronchoalveolar lavage fluid (BALF) was collected by washing three times with 1 ml of phosphate buffered saline (PBS) in the lung. BALF samples were centrifuged at 300 g for 5 min at 4 °C, and the cell pellets were re-suspended in 1 ml of PBS. The total live-cell count was performed using a hemocytometer. Then, the cells were cytospun into a microscope slide and stained with a Diff-Quick Staining kit (Thermo Fisher Scientific, Waltham, MA, USA). The BALF cells were obtained by counting leukocytes under light microscopy.

### Histopathological staining

Left lungs were detached from the mice and were immediately fixed with 4% paraformaldehyde before embedding with paraffin wax and routine processing. Serial paraffin sections (5 μm) were prepared using a microtome, and deparaffinized tissue sections were stained with hematoxylin and eosin (H&E, Sigma, St. Louis, USA) to evaluate morphological changes in lungs. The severity of lung fibrosis was evaluated using Masson’s trichrome staining (Sigma, St. Louis, USA). Masson’s staining was carried out in order to differentiate collagen from other fibers by staining nuclei in black, cytoplasm and muscles in red, and collagen in blue.

#### Histopathological scoring

To evaluate the score of pulmonary inflammation from H&E stained tissues, inflammation was quantified by a set of custom-designed criteria as described previously^40^. The score was based on 13-point scale, which evaluates airway inflammation (4 points), vascular inflammation (4 points), and parenchymal inflammation (5 points). All scoring was performed blinded and an average score was assigned to each lung.

#### Ashcroft scoring

To determine the severity of pulmonary fibrosis, each field was semi-quantitatively assessed, as described previously with minor modifications^41^. Criteria for grading fibrosis were as follows: Grade 0, normal lung; Grade 1, minimal fibrous thickening of alveolar or bronchiolar walls; Grade 2–3, moderate thickening of walls without obvious damage to lung architecture; Grade 4–5, increased fibrosis with definite damage to lung structure and formation of fibrous bands or small fibrous masses; Grade 6–7, severe distortion of structure and large fibrous areas (honeycomb lung is placed in this category); Grade 8, total fibrous obliteration of the fields.

#### Morphometric analysis of collagen

To quantify collagen deposition, we adopted a previously described image analysis-based system with minor modifications^42,43^. Briefly, images of Masson’s trichrome-stained specimens were captured with on a digital camera and digitalized at 2560 × 1920 pixel spatial resolution with a global magnification of 400. By adjusting image contrast and threshold, the images were processed to detect areas of blue-stained collagen on each field. The fractional area of fibrosis are given as proportion of collagen area of the total area (%). The average percent area of fibrosis was generated from five non-overlapping fields per section and assigned to each animal.

### Hydroxyproline assay

The collagen content in the lung was quantified by a hydroxyproline assay. 10 mg of lung tissue was hydrolyzed in 6N HCl at 120 °C for 3 h, followed by dried overnight. The samples were centrifuged 10,000 g for 20 min at 4 °C and the supernatants were collected. The hydroxyproline content was determined using a kit (Hydroxyproline Assay Kit, Sigma) according to the manufacturer’s instructions. The absorbance of each sample at 550 nm wavelength was measured using a standard curve (Hyproxyproline standards (0–10 μg/ml).

### Enzyme-linked immunosorbent assay

TNF-α, IL-6, IL-1β, TGF-β1, IL-17A, and IL-23 levels in lung homogenates were determined using a quantitative sandwich enzyme-linked immunosorbent assay (ELISA) according to the manufacturer’s instructions (BD sciences, San Diego, CA, USA). Briefly, right lung samples were homogenized in 50 mM potassium phosphate buffer containing protease inhibitors (Roche, Germany) and centrifuged at 12,000 rpm for 15 min at 4 °C. The supernatants were collected and stored −80 °C until use. OD (optical density) of each sample after color development was measured with a microplate reader (SOFT max PRO software, Sunnyvale, CA, USA) at 450 nm.

### Quantitative Real-time PCR

Total RNA was extracted from lung tissues and cells using Easy Blue^TM^ (Intron Company, Seongnam, South Korea). First-strand cDNA was synthesized with a cDNA synthesis kit (Bioneer Corporation, Daejeon, South Korea). Then, the mRNA levels of TGF-β1, E-cadherin, α-SMA, vimentin, Slug, Snail, and type I collagen were analyzed in a LightCycler 96 (Roche, Basel, Switzerland) with SYBR Green qPCR Mastermix (Bioline Reagents Ltd., London, United Kingdom) in a total volume of 20 μl Relative levels of mRNA expression were normalized to β-actin expression for each gene. Primers are listed in Supplementary Table 2.

### Western blotting

Samples of mice lung tissues and cells were homogenized in RIPA buffer (Cell Signaling, Boston, MA, USA) in the presence of a protease inhibitor cocktail (Roche, Basel, Switzerland). Approximately 30 μg of total protein was separated by SDS-PAGE, then transferred to a nitrocellulose membrane (EMD Millipore, Billerica, MA, USA). The membranes were blocked with 5% BSA (bovine serum albumin) for 0.5 h, followed by incubation with appropriate primary antibodies overnight at 4 °C. Then, after washes with TBST (20 mM Tris-HCl at pH 7.4, 150 mM NaCl, 0.05% Tween-20), the membranes were incubated with HRP-labeled secondary antibodies and visualized with enhanced chemiluminescence reagents (DoGen, Seoul, South Korea). The results were analyzed using the ImageJ densitometry system.

### Immunofluorescence staining

The cells were seeded on glass slides and treated as described above. Then, the samples were washed with PBS, fixed with 4% paraformaldehyde for 10 min and permeabilized with 0.2% Triton X-100 in PBS. After blocking with 5% BSA, the cells were stained with the appropriate primary antibodies at 4 °C overnight. After incubation, the samples were washed and incubated with Alexa Fluor 594- or 488-conjugated antibody (Life Technologies, USA). The nuclei were stained with DAPI. Then, the samples were washed with PBS and mounted with anti-fade solution (Vector Laboratories, Burlingame, CA, USA). Fluorescent images were acquired with a Zeiss LSM5 confocal microscope (Carl Zeiss, Jena, Germany).

### Wound healing assay

Wound healing assay as performed as previously described with the minor modification^27^. Briefly, A549 or HLF cells were grown on 6-well plates until they formed a confluent monolayer. A scratch was made using a sterile 200 μl micropipette tip and cells were washed with PBS and treated TGF-β1 or PM014. The cell migration was tracked over time and imaged by phase contrast microscopy after 48 hours of treatment. The scratch assay was performed on 4 replicate cultures for each condition. Each culture was imaged at different 3 location at 3 locations. The percentage of original width was estimated by measuring the width between the edges of the scratch would in the distinctive area of each image using Image J software.

### Statistical analysis

Data were expressed as the means ± SE. A one-way analysis of variance (ANOVA) followed by the Newman-Keuls test was performed. All experiments were performed with at least three biological replicates, and *P* < 0.05 was considered statistically significant.

## Electronic supplementary material

Supplementary information

## Data Availability

No datasets were generated or analyzed during the current study.
