# Supplementary material for: A standardized herbal extract PM014 ameliorates pulmonary fibrosis by suppressing the TGF-β1 pathway
Source: Sci Rep. 2018 Nov 15;8:16860. doi: 10.1038/s41598-018-35320-8 (PMC6237877; doi:10.1038/s41598-018-35320-8)
Supplement: Supplementary file 1 — Supplementary information [file 41598_2018_35320_MOESM1_ESM.pdf]

A standardized herbal extract PM014 ameliorates pulmonary fibrosis by suppressing the TGF-β1 pathway.

Kyung Hwa Kim[1], Sujin Lee[1], Hyunji Lee[1], Dasom Shin[1], Daeun Min[1], Miran Kim[2], Byeol Ryu[3], Hyun Woo Kim[3] & Hyunsu Bae[1,*]

[1] Department of Physiology, College of Korean Medicine, Kyung Hee University, Seoul 02447, South Korea
[2] Central Research Institute, Hanlim Pharm. Co. Ltd., Yongin 17040, South Korea
[3] College of Pharmacy and Research Institute of Pharmaceutical Science, Seoul National University, Seoul 08826, South Korea.

* Correspondence to Dr. Hyunsu Bae
Department of Physiology, College of Korean Medicine, Kyung Hee University, Seoul 02447, South Korea.
Tel: +82-2-961-9316
Email: hbae@khu.ac.kr
Fax: +82-70-4194-9316

Table S1. Composition of PM014.

Formula	Amount (g)	Bioactive constituent
Root of Rehmannia glutinosa (RG)	600	5-hydroxymethyl-2-furaldehyde
Root of Paeonia suffruticosa (PS)	300	Paeoniflorin
Fruit of Schisandra chinensis (SC)	300	Schizandrin
Root of Asparagus cochinchinensis (AC)	300	Asparagine
Seed of Prunus armeniaca (PA)	225	Amygdalin
Root of Scutellaria baicalensis (SB)	225	Baicalin
Root of Stemona sessilifolia (SS)	150	Stemonine
Total	2100	

Table S2. Primer sequences used for real-time qPCR.

Gene	Forward primer (5'-3')	Reverse primer (5'-3')
Mouse α-SMA	CTCTGCCTCTAGCACACAACT	GGCCAGGGCTACAAGTTAAGG
Mouse E-cadherin	CGTCTCCTCATGGCTTTGC	CTTTAGATGCCGCTTCAC
Mouse vimentin	CGGAAAGTGGAATCCTTGCA	CACATCGATCTGGACATGCTGT
Mouse β-actin	GATCTGGCACCACACCTTCT	GGGGTGTTGAAGGTCTCAAA
Mouse type I collagen	GGGTGAGACAGGCGAACAAG	AACCAGCAGAGCCAGGGG
Mouse TGF-β1	TACGTCAGACATTCGGGAAGCA	AGGTAACGCCAGGAATTGTTGC
Human TGF-β1	TGTGTGCTGAAGCCATCGTTG	CCGGCTTGTCTGAAAAGGTCA
Human E-cadherin	TGAGAACGAGGCTAACG	TCACATCCAGCACATCC
Human vimentin	AAAGTGTGGCTGCCAAGAACCT	ATTTCACGCATCTGGCGTTCCA
Human Slug	GAGCATTTGCAGACAGGTCA	ACAGCAGCCAGATTCCTCAT
Human Snail	GCCTAGCGAGTGGTTCTTCT	TAGGGCTGCTGGAAGGTAAA
Human type I collagen	GTTCGTGACCGTGACCTCG	TCTTGTCCTTGGGGTTCTTGC
Human β-actin	CCAACCGCGAGAAGATGA	CCAGAGGCGTACAGGGATAG

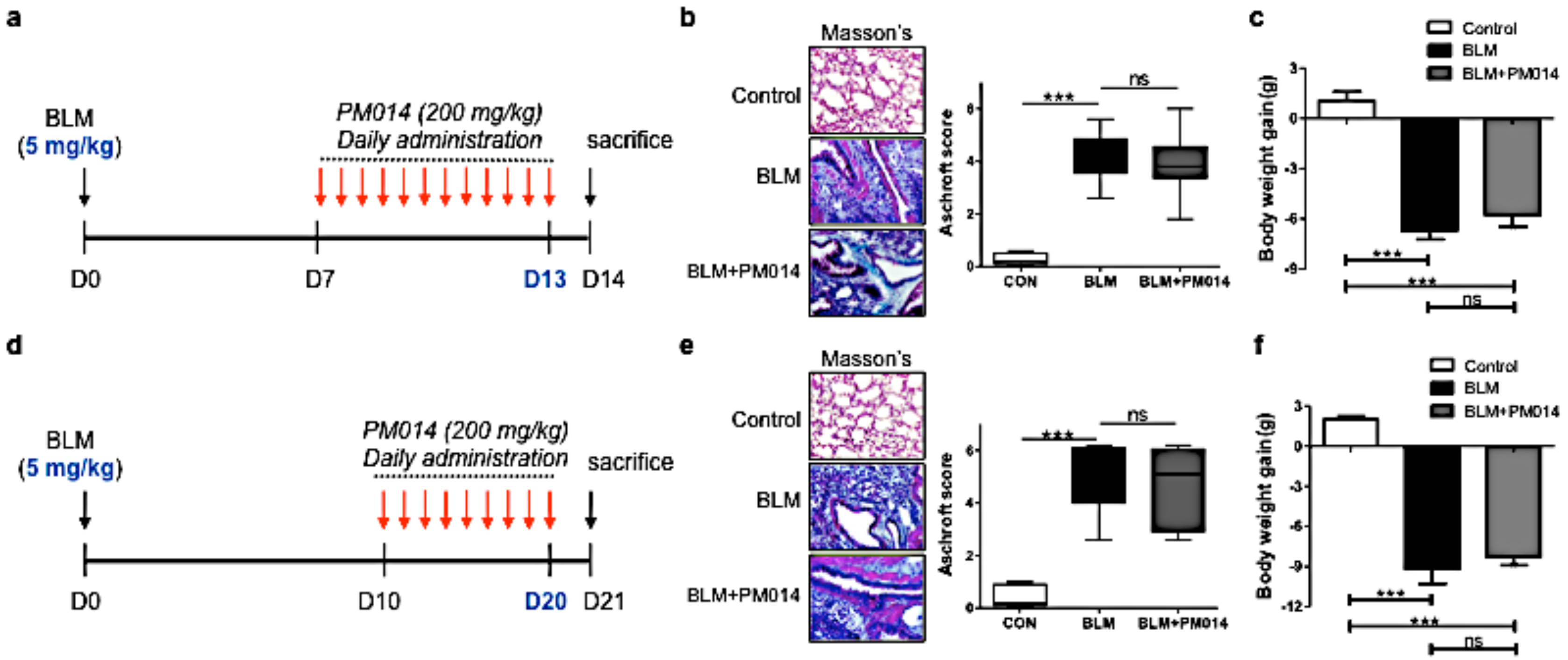

Fig. S1. PM014 exerts no significant anti-fibrotic effect in BLM-induced lung fibrosis when given during the fibrogenic phase.

Mice were administered with BLM (5 mg/kg) at day 0 and then treated once a day by PM014 (200 mg/kg) from day 7 to day 13 (a-c) or from day 10 to day 20 (d-f) after BLM challenge. (a,d) The experimental schedule was represented. (b,e) Representative images of Masson's trichrome staining were shown and Ashcroft scoring was carried out to determine the extent of lung fibrosis. (c,f) body weight gain over the experiments was calculated at day 13 (c) or day 20 (f) after BLM exposure. NS indicates no significant differences were found among groups. n = 5-9 for each experimental group, **P* < 0.05; ****P* < 0.001, Data are presented as mean ± SE.

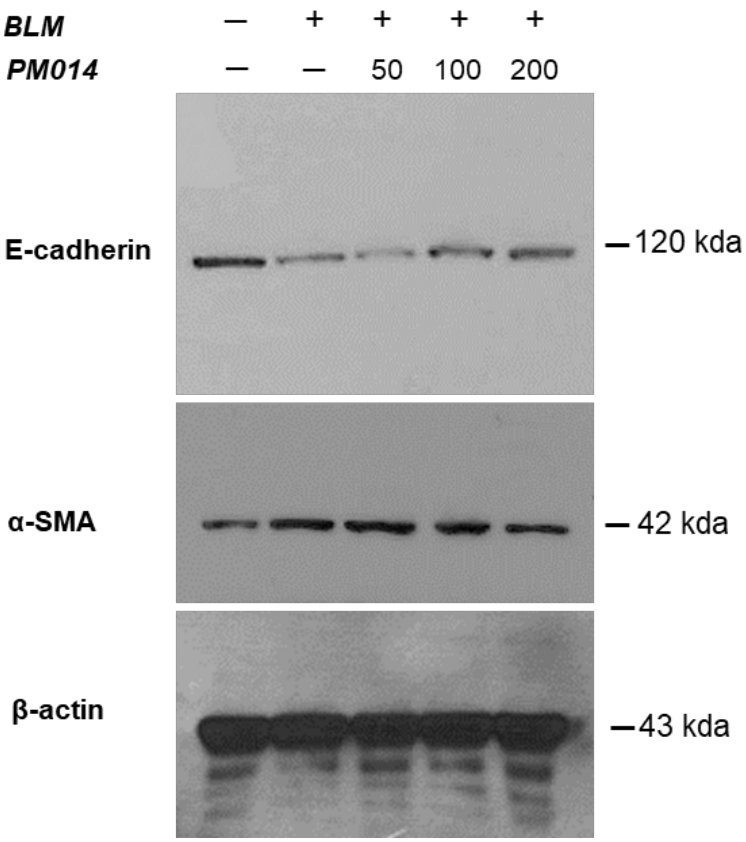

Fig. S2. Uncropped western blots from Fig. 4.

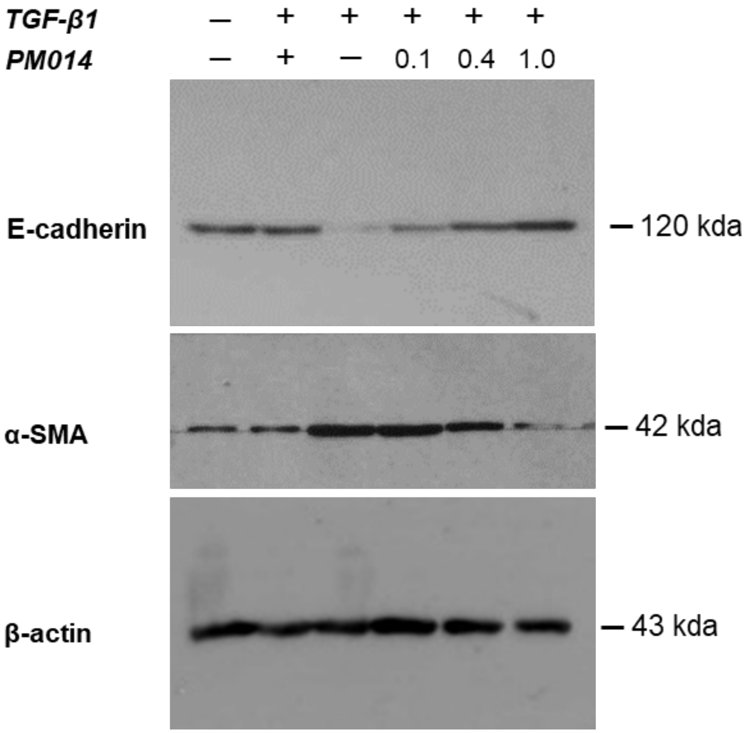

Fig. S3. Uncropped western blots from Fig. 5.

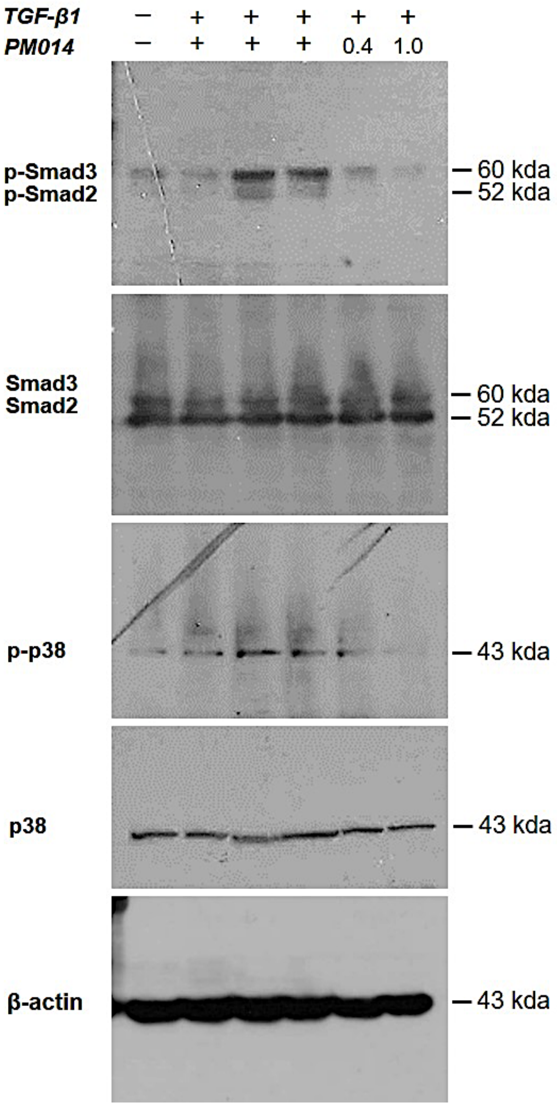

Fig. S4. Uncropped western blots from Fig. 6.

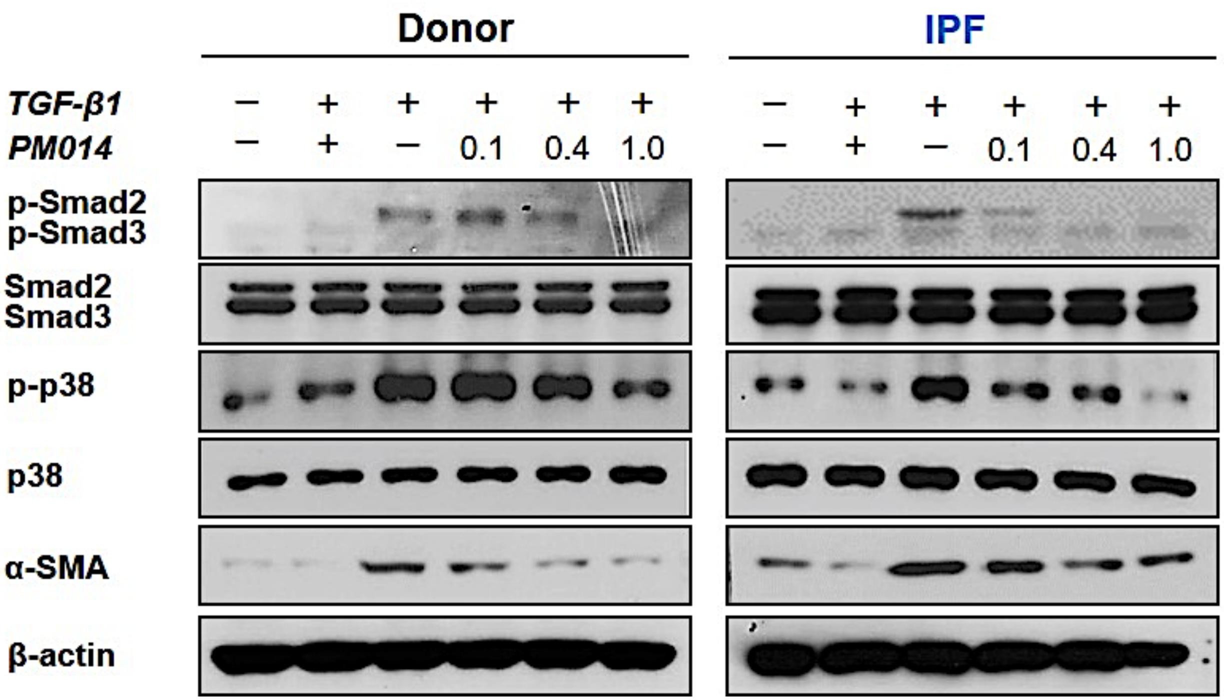

Fig. S5. PM014 targets TGF-β1 signaling pathway in EMT stimulated by TGF-β1 treatment on human lung fibroblasts.

Healthy donor or IPF human lung fibroblasts (HLFs) were stimulated with TGF-β1 (20 ng/ml) and PM014 (0.1, 0.4, 1.0 mg/ml) for 48 h. The phosphorylation levels and total levels of Smad2/3 and p38 MAPK were measured by western blot analysis. The expression level of mesenchymal marker α-SMA was assessed to confirm EMT phenotypes after TGF-β1 in fibroblasts. β-actin was used as the protein loading control. Shown are the representative images of three independent experiments.

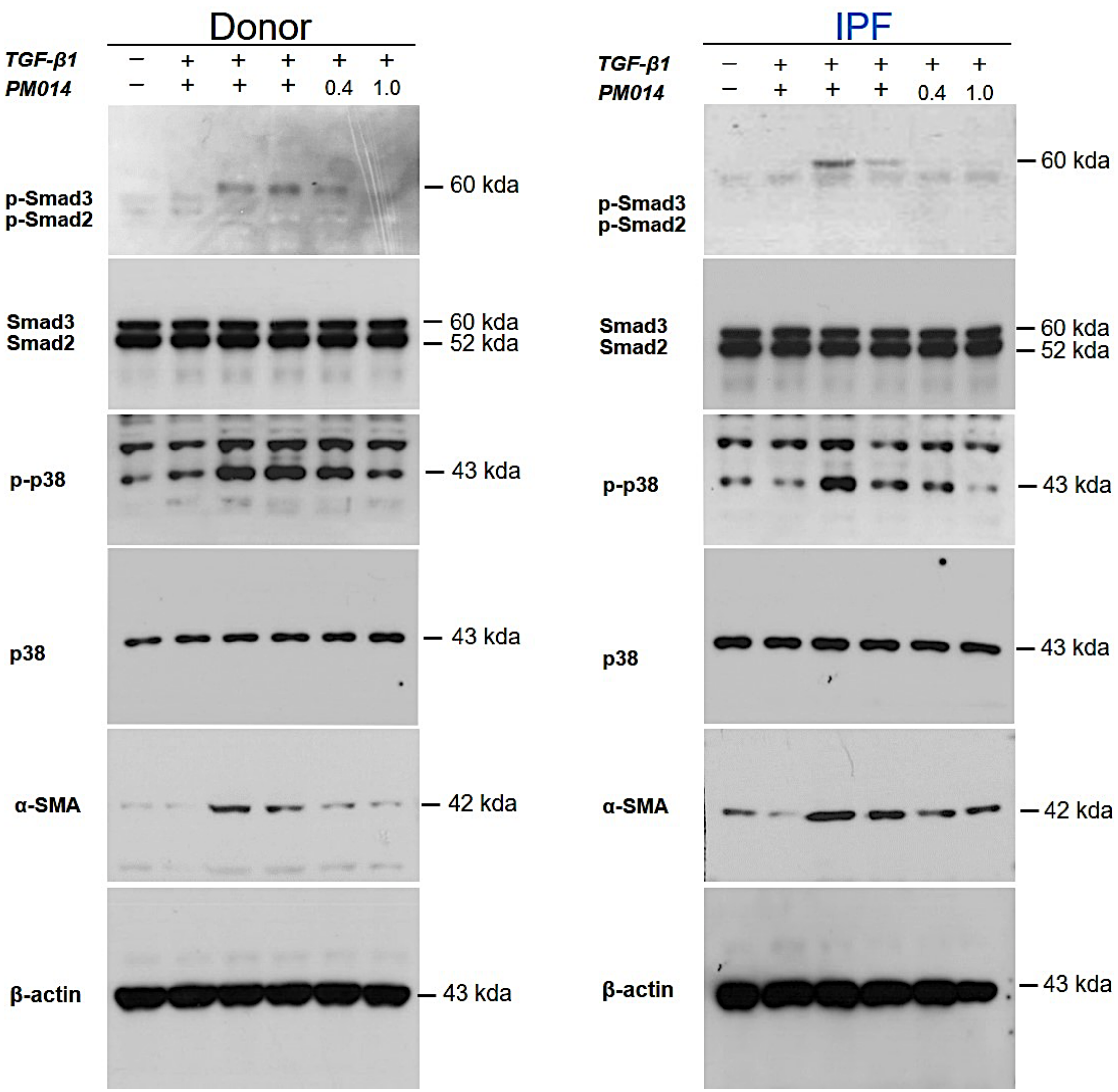

Fig. S6. Uncropped western blots from Fig. S5.

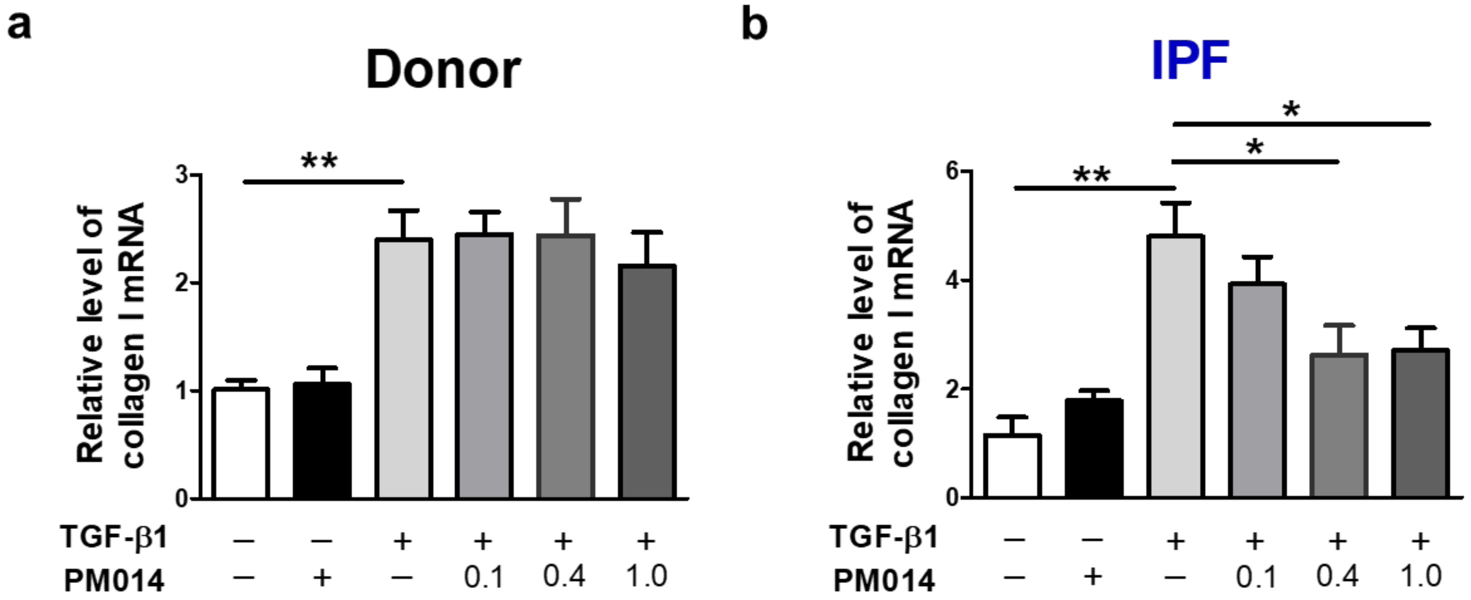

Fig. S7. PM014 reduces TGF-β1-induced increase of collagen in human lung fibroblasts.

Healthy donor (a) or IPF (b) human lung fibroblasts (HLFs) were stimulated with TGF-β1 (20 ng/ml) and PM014 (0.1, 0.4, 1.0 mg/ml) for 48 h. mRNA level of type I collagen was measured by real-time qPCR analysis. Expression of mRNA was normalized to expression of β-actin. Values are mean ± SE of results from independent 4 biological replicates, *P < 0.05; **P < 0.01.

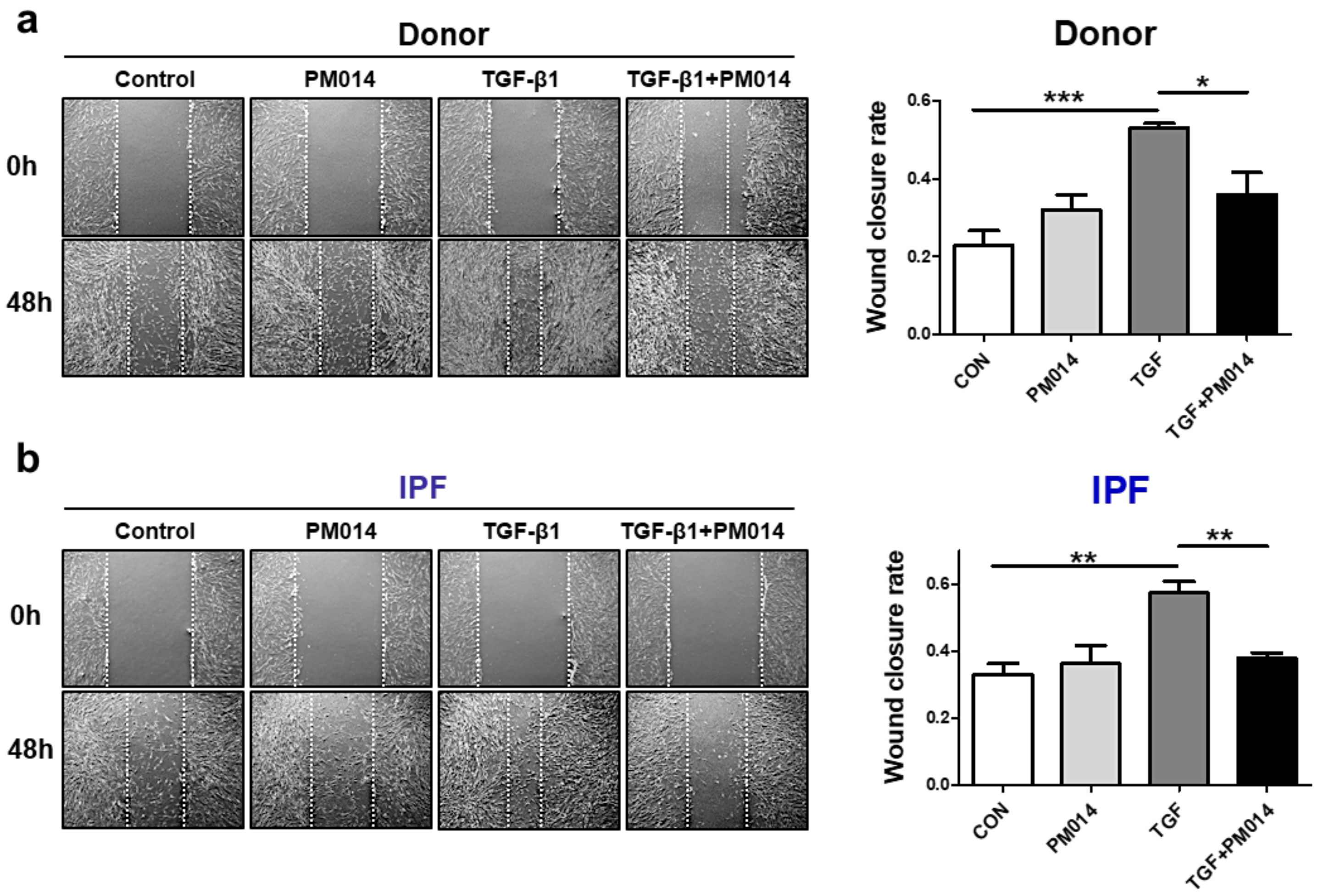

Fig. S8. PM014 inhibits cell migration in EMT induced by TGF-β1 in human lung fibroblasts.

Human lung fibroblasts from Healthy donor (a) or IPF patient (b) were treated with TGF-β1 (20 ng/ml) and PM014 (0.4 mg/ml) for 48 h. The wound closure rate, representing the migration rate, was analyzed. Values are mean ± SE. n = 4, **P* < 0.05; ***P* < 0.01; ****P* < 0.001.
